# A Novel Mechanism of Ataxia Telangiectasia Mutated Mediated Regulation of Chromatin Remodeling in Hypoxic Conditions

**DOI:** 10.3389/fcell.2021.720194

**Published:** 2021-09-21

**Authors:** Maria Likhatcheva, Roben G. Gieling, James A. L. Brown, Constantinos Demonacos, Kaye J. Williams

**Affiliations:** ^1^Division of Pharmacy and Optometry, Faculty of Biology Medicine and Health, School of Health Science, University of Manchester, Manchester, United Kingdom; ^2^Department of Applied Sciences, Northumbria University, Newcastle upon Tyne, United Kingdom; ^3^Department of Biological Science, University of Limerick, Limerick, Ireland; ^4^Discipline of Biochemistry, Centre for Chromosome Biology, School of Science, National University of Ireland Galway, Galway, Ireland; ^5^Health Research Institute, University of Limerick, Limerick, Ireland

**Keywords:** Ataxia Telangiectasia Mutated (ATM), hypoxia, SUV39H1, Tip60, MDM2

## Abstract

The effects of genotoxic stress can be mediated by activation of the Ataxia Telangiectasia Mutated (ATM) kinase, under both DNA damage-dependent (including ionizing radiation), and independent (including hypoxic stress) conditions. ATM activation is complex, and primarily mediated by the lysine acetyltransferase Tip60. Epigenetic changes can regulate this Tip60-dependent activation of ATM, requiring the interaction of Tip60 with tri-methylated histone 3 lysine 9 (H3K9me3). Under hypoxic stress, the role of Tip60 in DNA damage-independent ATM activation is unknown. However, epigenetic changes dependent on the methyltransferase Suv39H1, which generates H3K9me3, have been implicated. Our results demonstrate severe hypoxic stress (0.1% oxygen) caused ATM auto-phosphorylation and activation (pS1981), H3K9me3, and elevated both Suv39H1 and Tip60 protein levels in FTC133 and HCT116 cell lines. Exploring the mechanism of ATM activation under these hypoxic conditions, siRNA-mediated Suv39H1 depletion prevented H3K9me3 induction, and Tip60 inhibition (by TH1834) blocked ATM auto-phosphorylation. While MDM2 (Mouse double minute 2) can target Suv39H1 for degradation, it can be blocked by sirtuin-1 (Sirt1). Under severe hypoxia MDM2 protein levels were unchanged, and Sirt1 levels depleted. SiRNA-mediated depletion of MDM2 revealed MDM2 dependent regulation of Suv39H1 protein stability under these conditions. We describe a novel molecular circuit regulating the heterochromatic state (H3K9me3 positive) under severe hypoxic conditions, showing that severe hypoxia-induced ATM activation maintains H3K9me3 levels by downregulating MDM2 and preventing MDM2-mediated degradation of Suv39H1. This novel mechanism is a potential anti-cancer therapeutic opportunity, which if exploited could target the hypoxic tumor cells known to drive both tumor progression and treatment resistance.

## Introduction

The genome is constantly exposed to exogenous and endogenous factors that can affect its function and stability. One of the most important cellular mechanisms that safeguards genome integrity is the DNA damage response pathway (DDR) (Shiloh, 2003; Ciccia and Elledge, 2010). DDR is a chromatin-associated process that is activated in response to different types of cellular stress. One of the key factors of DDR is the phosphatidylinositol-3-kinase (PI3K)—like kinase Ataxia Telangiectasia Mutated (ATM) (Zou and Elledge, 2003; Ciccia and Elledge, 2010).

The genome, through chromatin structure, is regulated by posttranscriptional modifications (PTM) of histones, including phosphorylation, methylation and acetylation (Cedar and Bergman, 2009; Bannister and Kouzarides, 2011; Zhou et al., 2011). Heterochromatic DNA is characterized by the presence of tri-methylation of lysine 9 of histone 3 (H3K9me3). ATM is essential for the repair of DNA double strand breaks (DSB) in the heterochromatic region of the genome (Nakayama et al., 2001; Di Micco et al., 2006; Goodarzi et al., 2008). Following DSB, ATM is activated by trans auto-phosphorylation at S1981 forming active ATM monomers. This event is mediated by lysine acetyltransferase Tip60-dependent acetylation of ATM (Sun et al., 2005, 2007). Additionally, DSB induce direct interactions of Tip60 with the H3K9me3 (Sun et al., 2009). ATM can be activated (independent of DDR signaling) in response to hypotonic stress, chromatin modifying agents, heat shock and hypoxia (Hunt et al., 2007; Kanu and Behrens, 2007; Guo et al., 2010). Hypoxia induced ATM activation has been associated with stalled replication forks, H3K9me3 and DDR gene expression (including BRCA1 and MLH1) (Bencokova et al., 2009; Lu et al., 2011, 2014; Olcina et al., 2013, 2015). Under hypoxic conditions Tip60 is catalytically active (Perez-Perri et al., 2016). However, it is unknown if hypoxia-induced ATM activation remains Tip60-dependent. Interestingly, inducing chromatin relaxation using histone deacetylase inhibitors (HDACi) increases DDR signaling, apoptosis and tumor regression *in vivo* (Di Micco et al., 2011).

Hypoxia is a common feature of most solid tumors and is associated with poor prognosis, a more aggressive tumor phenotype, and radio- and chemo-resistance (Bertout et al., 2008). The cellular adaptation to hypoxic stress alters the histone epigenetic profile, contributing to tumorigenic genomic instability and resistance to therapy (Falk et al., 2008; Watson et al., 2009, 2010; Krieg et al., 2010; Perez-Perri et al., 2011; Takata et al., 2013; Allshire and Madhani, 2018). Recently, H3K9me3 was identified as the most efficient barrier to cellular reprogramming, preventing cellular dedifferentiation (Jehanno et al., 2017). H3K9me3 is catalyzed predominantly by the ubiquitously expressed methyltransferase Suv39H1 (Rea et al., 2000; Hagemann et al., 2000). Aberrant Suv39H1 expression has been reported in a number of solid tumors (Ozdağ et al., 2006). The protein levels of Suv39H1 are regulated by posttranslational modifications (Bosch-Presegue et al., 2011; Mungamuri et al., 2016; Kim et al., 2021) and the ubiquitin E3 ligase murine double minute 2 (MDM2) (Bosch-Presegue et al., 2011; Mungamuri et al., 2016). It has been shown that Suv39H1 promotes heterochromatin formation in response to different types of stress, including ionizing radiation (IR) (Bosch-Presegue et al., 2011; Wang et al., 2013; Sidler et al., 2014). However, little is known about Suv39H1 regulation in response to hypoxic stress.

It has been demonstrated that hypoxia induces a global increase in H3K9 methylation in cancer cell lines (Chen et al., 2006; Tausendschon et al., 2011; Olcina et al., 2013). Suv39H1 induction in response to hypoxia has been correlated with the levels of H3K9me3 in human fetal lung epithelial cells (Benlhabib and Mendelson, 2011) as well as in mouse embryonic fibroblasts (MEFs) (Olcina et al., 2013). However, its role in regulating H3K9me3 in hypoxic cancer cells is unknown, and the molecular network(s) orchestrating potential correlations have not been elucidated.

Hypoxia is known to cause ATM activation that is independent of DNA damage (Olcina et al., 2013). Additionally, ATM has been implicated in suppressing MDM2 function (Gannon et al., 2012). However, whether these events coincide in hypoxia is currently unclear. Considering that the levels of Suv39H1 are regulated by MDM2 in normoxia, we propose that the same mechanism is operating in response to hypoxic stress. As such, the induction of ATM followed by MDM2 inactivation in hypoxia might lead to increased levels of Suv39H1 triggering H3K9me3. In this study the molecular mechanism regulating Suv39H1 stability and the subsequent induction of H3K9me3 were investigated. The effects of the ATM mediated regulation of MDM2 on Suv39H1 were monitored in hypoxia. The results support the view of the existence of a regulatory mechanism of chromatin remodeling under hypoxic conditions involving activation of ATM. This novel ATM dependent mechanism for the maintenance of the heterochromatic state in hypoxic conditions indicates that chromatin-modifying drugs targeting ATM function could be exploited to provide therapeutic benefits to late-stage tumors.

## Materials and Methods

### Cell Line and Reagents

HCT116 (colon carcinoma, p53 wild type) and FTC133 (Human follicular thyroid carcinoma, mutated p53) were grown in RPMI-1640 or DMEM media combined with HAM’s F12 (1:1) respectively (Sigma-Aldrich, Poole, Dorset, United Kingdom). The media was supplemented with 10% (v/v) FBS (GIBCO PRL, Paisley, United Kingdom). Cell culture was performed using a class II laminar flow microbiological safety cabinet. Cells were treated with 10 μM of Ku55933 for 6 h. Cells were radiated with 4 Gy using a Faxitron X-ray (Faxitron Bioptics, AZ, United States). Cells were grown in a humidified incubator at 37°C supplied with 5% CO_2_. Mycoplasma testing was carried out periodically using core facilities at The University of Manchester. All cell lines were obtained from ATCC and authenticated using service provided by Public Health England (last tested in September 2019, prior to completion of these studies).

### Hypoxic Conditions

A Whitley H35 Hypoxystation (Don Whitley Scientific Limited, Shipley, United Kingdom) was used in order to create the hypoxic condition used. All experiments were carried out under severe hypoxic conditions (0.1% O_2_). Cells were seeded and allowed to adhere to the cell culture dish overnight before being transferred to the hypoxic chamber. Cells were incubated for 6 or 18 h in hypoxia and lysed inside the hypoxic chamber.

### Western Blot

Cells were lysed in RIPA buffer (Tris-HCl 50 mM at pH 7.4, NaCl 150 mM, IGEPAL 1%, EDTA 1 mM) with phosphatase inhibitors (PMSF 1 mM, Na3VO4 1 mM and NaF 1 mM), sonicated and centrifuged for 10 min at 14 000 *g* and the insoluble debris was discarded. Cell lysate (10–35 μg of protein) was fractionated by gel electrophoresis using precast NuPAGE^TM^ gels (Invitrogen, Paisley, United Kingdom) and transferred to a PVDF membrane (BioRad, Hertfordshire, United Kingdom). The membrane was blocked for 1h with Tris Buffered Saline (Sigma Aldrich, United Kingdom) containing 5% non-fat dry milk and 0.1% Tween 20, incubated with primary and secondary antibodies, and the membrane was developed using enhanced chemiluminesce (ECL) substrate (Bio-Rad). H3K9me3, H3, MDM2, p53, ATM and ATM-pSer1081 were detected using antibodies from Abcam (Cambridge, United Kingdom). HIF-1α was detected using an antibody from BD Transduction. Anti-SUV39H1 and anti-Sirt1 antibody was from Millipore (Billerica, MA, United States). Anti- actin was from Santa Cruz Biotechnology (Santa Cruz, CA, United States). The specificity of two different MDM2 antibodies (anti-MDM2 EP16627 and anti-MDM2 2A10) were validated in FTC133 cells treated with MDM2 siRNA (Supplementary Figure 1).

### Immunofluorescence Staining

Cells cultured onto a sterile coverslip were fixed using 10% formalin in PBS, blocked with 1% (w/v) BSA in PBS for 30 min and incubated with anti-H3K9me3 in blocking buffer for 1 h. Cover-slips were washed with PBS + 0.1% Triton X-100, incubated with anti-rabbit AlexaFluor 488. Microscopy images were collected on a Zeiss Axio Imager.D2 upright microscope using a 40x/0.5 EC Plan-neofluar objective and captured using a Coolsnap HQ2 camera (Photometrics) through Micromanager software v1.4.23. Specific band pass filter sets for DAPI and FITC were used to prevent bleed through from one channel to the next. Images were then processed and analyzed using Fiji ImageJ software.

### RNA Isolation and Quantitative PCR

RNA was extracted using the RNasey kit (Qiagen, Manchester, United Kingdom). cDNAs were prepared by reverse transcription of total RNA using High Capacity cDNA Reverse Transcription Kit (Thermo Fisher Scientific, United Kingdom). The products were used for real-time PCR using TaqMan probes for Suv39H1, CA9, MDM2, HRT1 and Actin-β (Dharmacon, Horizon Discovery, Cambridge, United Kingdom). RT-PCR was performed using TaqMan Fast Advance master mix (Thermo Fisher Scientific, United Kingdom) in a StepOnePlus RT-qPCR instrument (Thermo Fisher Scientific, United Kingdom). The obtained data were analyzed using ΔΔC_*q*_ method to quantify the relative gene expression as described in Livak and Schmittgen (2001).

### RNA Interference

Cells were transfected with control small interfering RNA (sc-37007), SMARTpool MDM2 siRNA (SO-2650613G), or Suv39H1 siRNA (Cy5GGUGAAAUGGCGUGGAUAUUU3′) from Dharmacon using lipofectamine 2000 (Invitrogen), according to instructions from the supplier. Cells were treated and analyzed after a total of 72 h post transfection.

### Ataxia Telangiectasia Mutated Inhibition

Cells were treated with 10 μM of Ku55933 for 6 h before lysis (Hickson et al., 2004).

### Statistical Analysis

Statistical analysis was carried out using Graphpad Prism version 7, once the data had been repeated at least three times. When comparing data obtained from experiments with only two different conditions (e.g., normoxia vs. hypoxia) an unpaired *t*-test was used to compare treated and untreated data. When comparing data obtained from experiments with more than one variable (e.g., normoxia with or without drug vs. hypoxia with or without drug) analysis of variance (ANOVA) was used, and to identify individual differences Sidak’s multiple comparisons test was performed. The obtained *P*-values are represented as follows: a *p-*value of ≤ 0.05 is represented as ^∗^, a *p*-value of ≤ 0.01 is represented ^∗∗^, a *p*-value of ≤ 0.001 is represented ^∗∗∗^ and a *P*-value of ≤ 0.0001 is represented as ^****^.

## Results

### Ataxia Telangiectasia Mutated Activation in Response to Hypoxia Coincides With Upregulation of Suv39H1 and H3K9me3

**FIGURE 1:**
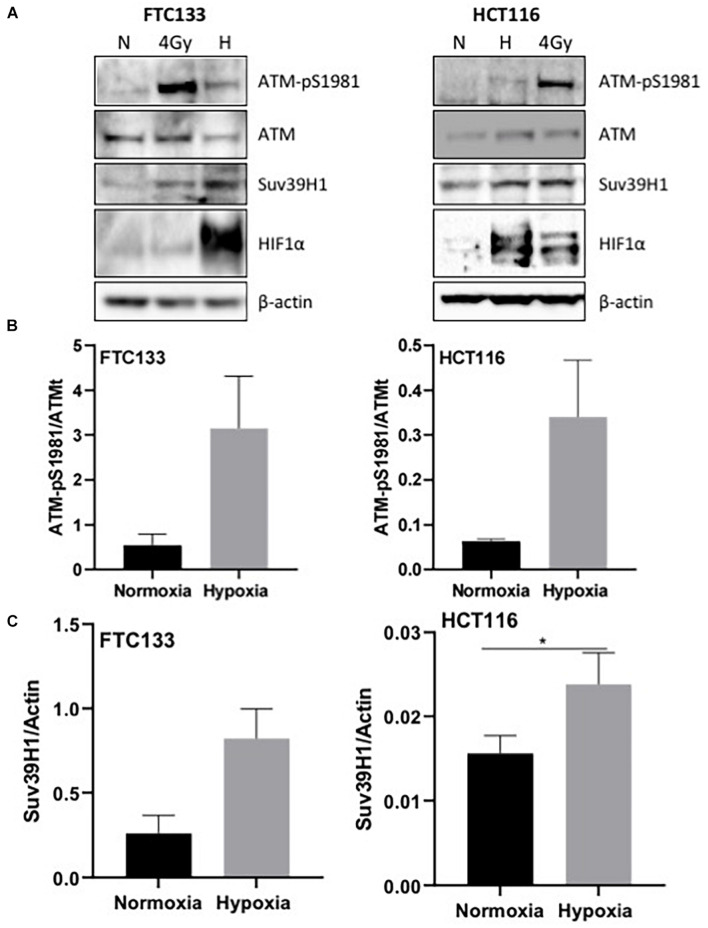
pATM-S1981, Suv39H1 and H3K9me3 are upregulated in response to hypoxia. Cells were incubated in normoxic (N; 21% O_2_) or hypoxic conditions (H; 0.1% O_2_) for 18 h prior to lysis and Western blotting **(A)**. The graph represents the protein levels of ATM-pS1981 **(B)** and Suv39H1 **(C)** normalized to the loading control. HIF-1α was used as a control for hypoxia and β-actin as a loading control. Three independent experiments were performed and the bar represents the mean ± SEM. *p ≤ 0.05.

**FIGURE 2:**
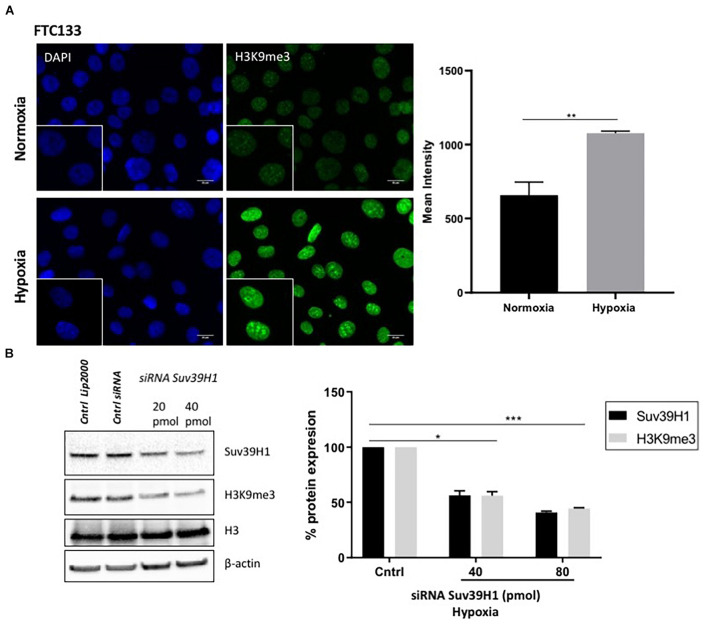
H3K9me3 upregulation in hypoxia is Suv39H1 dependent. FTC133 cells were incubated for 18 h in normoxic (21% O_2_) or hypoxic (0.1% O_2_) conditions then fixed and stained for H3K9me3 (green) and DAPI (blue) **(A)**. The graph represents H3K9me3 mean fluorescence intensity for each condition. FTC133 cells were transfected with Suv39H1 siRNA or control siRNA and incubated in hypoxia for 18 h. Cells were lysed and analyzed by Western blot. Densitometry analysis of Suv39H1 and H3K9me3 protein level is represented in the graphs as a percentage of protein expression by standardizing the levels of Suv39H1 with β-actin and control siRNA and the levels of H3K9me3 with the total amount of H3 and the control siRNA **(B)**. Three independent experiments were performed and the bar represents the mean ± SEM. *p ≤ 0.05, **p ≤ 0.01, ***p ≤ 0.001.

ATM activation in response to hypoxia has been previously reported (Bencokova et al., 2009). Here we followed ATM activation, indicated by ATM-pS1981 (pATM), in response to hypoxia (18 h, 0.1% O_2_) in two different cancer cell lines (human follicular thyroid carcinoma cells FTC133 and human colorectal carcinoma HCT116 cells) and this was compared to ATM auto-phosphorylation in normoxia (21% O_2_) (Figure 1A). Cells irradiated at 4Gy were used as a positive control for ATM activation. Consistent with previously reported data, active pATM was observed in hypoxic conditions (Figure 1B), and this ATM activation was independent of DNA damage (Supplementary Figure 2). An upregulation of Suv39H1 protein levels was also evident in hypoxia (Figure 1C). Since ATM activation is associated with H3K9me3, the H3K9me3 levels were analyzed using immunofluorescence in normoxic and hypoxic conditions. Significantly higher H3K9me3 protein levels were observed in FTC133 cells following hypoxic treatment compared to normoxia (Figure 2A), in accordance with previously published reports (Olcina et al., 2013, 2015). Upregulation of Suv39H1 in hypoxia coincided with higher H3K9me3 protein levels suggesting that Suv39H1 may mediate H3K9 trimethylation under these conditions (Hagemann et al., 2000; Rea et al., 2000). To assess whether Suv39H1 was involved in the upregulation of H3K9me3, siRNA-Suv39H1 or scrambled siRNA were transfected in FTC133 cells and the H3K9me3 protein levels were followed in the presence or absence of Suv39H1 expression (Figure 2B). Transient Suv39H1 knockdown in FTC133 cells in hypoxic conditions resulted in the downregulation of H3K9me3 (Figure 2B). Taken together, these results suggest Suv39H1 is involved in catalyzing H3K9me3 in hypoxia.

### Suv39H1 Is Regulated at the Protein Level in Hypoxia

**FIGURE 3:**
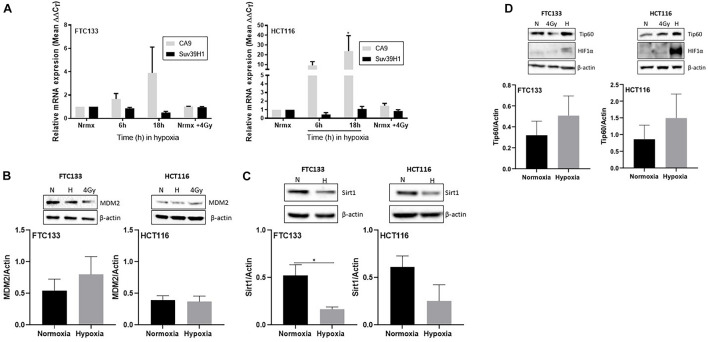
Suv39H1 is regulated at the protein level in hypoxia in a manner independent of MDM2 or Sirt1 protein expression levels. Cells were exposed for 6 or 18 h to normoxia (21% O_2_) or hypoxia (0.1% O_2_). After that time RNA was extracted and analyzed by RT-PCR. The graph represents the relative gene expression of Suv39H1 and CA9 compared to the normoxic control. CA9 is used as a control for hypoxia. Bars represent the mean ± SEM of four independent experiments **(A)**. Cells were incubated in normoxia (N; 21% O_2_) or severe hypoxia (H; 0.1% O_2_) for 18 h prior to lysis and Western blotting. HIF-1α was used as a control for hypoxic conditions and β-actin as a loading control. A representative image of one membrane is shown for MDM2 **(B)**, Sirt1 **(C)** and Tip60 **(D)**. The graphs represent the densitometry of protein levels normalized by the loading control. Three independent experiments were performed and the bar represents the mean ± SEM. *p ≤ 0.05.

To investigate the molecular mechanisms mediating Suv39H1 upregulation in hypoxia, Suv39H1 mRNA levels were investigated. No significant changes in Suv39H1 mRNA levels were detected following either 6 or 18 h hypoxic treatment (Figure 3A). In contrast, the mRNA expression of the known HIF-1α downstream target CA9 (Wenger et al., 2005) increased in a time dependent manner (Figure 3A). This indicates that under hypoxic conditions the upregulation of Suv39H1 level is a result of a mechanism regulating its protein stability rather than its gene expression.

Existing literature suggests that the E3-ubiquitin ligase MDM2 regulates Suv39H1 protein stability in normoxia (Bosch-Presegue et al., 2011; Mungamuri et al., 2016). However, to the best of our knowledge, the mechanism regulating Suv39H1 protein stability in hypoxic conditions is unknown. Considering the involvement of MDM2 in Suv39H1 regulation in normoxia, we hypothesized that a similar mechanism exists under hypoxic conditions. To test this hypothesis MDM2 protein levels were recorded in FTC133 and HCT116 cells following 18 h hypoxia (compared to normoxia). No significant changes in the MDM2 protein levels were evident in response to hypoxia (Figure 3B), suggesting the existence of a more complex system preventing MDM2 dependent degradation of Suv39H1 in hypoxia. Sirt1 has been shown to increase the half-life of Suv39H1 by inhibiting MDM2 mediated polyubiquitination in response to oxidative stress (Bosch-Presegue et al., 2011). To assess whether this mechanism was present under hypoxic conditions, Sirt1 protein levels were analyzed in FTC133 and HCT116 cells in normoxic and hypoxic conditions. Decreased Sirt1 protein levels were observed in hypoxic compared to normoxic conditions in both cell lines (Figure 3C). This suggests that Sirt1 is not involved in inhibiting MDM2 activity in hypoxia.

### Tip60 Is Involved in Maintaining Ataxia Telangiectasia Mutated Activation in Hypoxia

**FIGURE 4:**
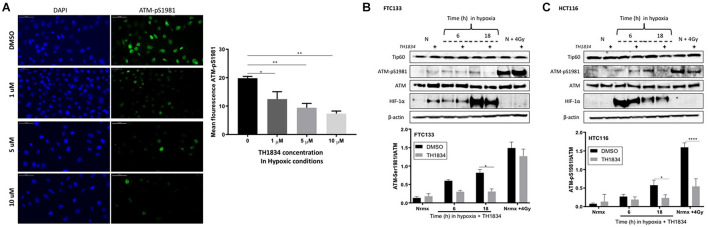
Tip60 mediates ATM auto-phosphorylation in hypoxia. FTC133 cells were incubated with 1, 5 or 10 μM of TH1834 or DMSO for 18 h in hypoxia (0.1% O_2_). Cells were stained for ATM-pSer1981 (green) and DAPI (blue). The graph represents ATM-pSer1981 mean fluorescence intensity **(A)**. FTC133 **(B)** and HCT116 **(C)** cells were incubated with or without 10 μM of TH1834 in normoxia (21% O_2_) or hypoxia (0.1% O_2_) for 6 or 18 h prior to lysis and Western blotting. Treatment with TH1834 is indicated with the symbol (+). Normoxia is indicated with an N and cells irradiated with 4 Gy as N + 4 Gy. HIF-1α was used as a control for hypoxia and β-actin as a loading control. The graphs represent the densitometry of ATM-pSer1981 protein level standardized to the total amount of ATM and the loading control in the presence of TH1834 (gray) or DMSO (black). Three independent experiments were performed and the bar represents the mean ± SEM. *p ≤ 0.05, **p ≤ 0.01, ****p ≤ 0.0001.

The role of Tip60 in regulating cellular responses to hypoxic stress has previously been highlighted (Perez-Perri et al., 2016). It is known that Sirt1 negatively regulates Tip60 protein levels and enzymatic activity (Wang and Chen, 2010; Peng et al., 2012) as well as the interaction of Tip60 chromodomain with H3K9me3 (Sun et al., 2009). We investigated the Tip60 protein levels in FTC133 and HCT116 cells in normoxic and hypoxic conditions (Figure 3D). An inverse correlation between Sirt1 (Figure 3C) and Tip60 (Figure 3D) protein levels was observed in response to hypoxia (Sirt1 downregulation and concomitant Tip60 upregulation). These results in combination with those shown in Figure 1A (ATM autophosphorylation in hypoxia) and Figure 2A (upregulation of H3K9me3 protein levels in hypoxic conditions) led to the hypothesis that ATM activation is Tip60-dependent in hypoxia. To test this hypothesis the activation of ATM was studied in hypoxic FTC133 and HCT116 cells treated with TH1834, a specific inhibitor of Tip60 acetyltransferase activity (Gao et al., 2014). The results showed a significant reduction of pATM levels in a TH1834 dose dependent manner in FTC133 cells (Figure 4A). Reduced pATM protein levels were observed in both FTC133 and HCT116 hypoxic cells (18 h) treated with TH1834 (Figures 4B,C, lanes 5 and 6). Since Tip60 activity depends on H3K9me3 (Sun et al., 2005, 2009), the mechanism governing H3K9me3 upregulation in hypoxic conditions was investigated next. Irradiated cells at 4 Gy were used as a positive control for Tip60 dependent activation of ATM in response to DNA damage (Sun et al., 2005, 2007). Surprisingly, FTC133 cell lines required higher concentrations of TH1834 to inhibit ATM in response to IR (Supplementary Figure 3). FTC133 cell lines present higher protein levels of ATM than HCT116, which may explain the observed difference.

### Ataxia Telangiectasia Mutated Dependent Inhibition of Mouse Double Minute 2 Leads to Suv39H1 Upregulation in Hypoxia

**FIGURE 5:**
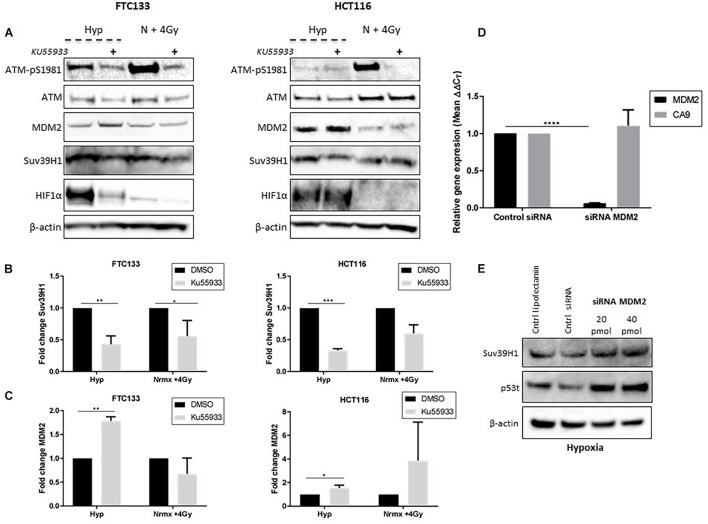
ATM dependent inhibition of MDM2 leads to Suv39H1 upregulation in hypoxia. Cells were incubated in normoxic (N; 21% O_2_) or hypoxic conditions (H; 0.1% O_2_) for 18 h. The ATM specific inhibitor (Ku55933) was added 6 h prior to lysis and Western blotting. Treatment with Ku55933 is indicated with the symbol (+). HIF-1α was used as a control for hypoxia and β-actin as a loading control. Cells irradiated with 4 Gy in normoxia and harvested 45 min later (N + 4 Gy) were used as a positive control for ATM activity **(A)**. Suv39H1 **(B)** and MDM2 **(C)** protein levels were calculated by densitometry and normalized to β-actin and untreated control for each condition. FTC133 cells were transfected with MDM2 siRNA or control siRNA and exposed for 18 h to hypoxia (0.1% O_2_). Total RNA was extracted and analyzed by RT-PCR. The graph represents the relative gene expression of MDM2 and CA9 compared to the control siRNA **(D)**. FTC133 cells transfected with 20, 40 pmol of MDM2 siRNA or control siRNA (Cntrl siRNA) were incubated in hypoxia (0.1% O_2_) for 18 h and the protein levels of Suv39h1 and p53 were analyzed by Western blot **(E)**. Three independent experiments were performed and the bar represents the mean ± SEM. *p ≤ 0.05, **p ≤ 0.01, ***p ≤ 0.001, ****p ≤ 0.0001.

ATM activation in response to severe hypoxia (≤ 0.1% O_2_), and ATM-mediated downregulation of MDM2 activity has been reported (Cheng et al., 2009; Gannon et al., 2012). We hypothesized that Suv39H1 protein stabilization under hypoxic conditions could be a consequence of ATM mediated MDM2 inhibition. To test this hypothesis the Suv39H1 protein levels were followed in FTC133 and HCT116 cells in which ATM was activated by hypoxia or IR (4 Gy), in the presence or absence of the ATM inhibitor Ku55933 (Hickson et al., 2004; Figure 5A). Significant downregulation of Suv39H1 was observed in hypoxic conditions upon treatment with Ku55933 in both cell lines (Figure 5B). Furthermore, significantly reduced Suv39H1 protein levels were seen under normoxic conditions in irradiated FTC133 cells treated with Ku55933 (Figure 5B). No effect of Ku55933 treatment in normoxic conditions on Suv39H1 protein levels was evident in FTC133 cells. However, in normoxic HCT116 cells treated with Ku55933 reduced Suv39H1 protein levels were observed as well as an increase in MDM2 protein levels (Figure 5C). It is important to note that the basal pATM levels in untreated HCT116 is higher than in FTC133 which may explain the observed difference between the two cell lines (Supplementary Figure 4).

Interestingly, the inhibition of ATM (directly by Ku55933, or indirectly by TH1834), led to the downregulation of HIF-1α in FTC133 cells (Figures 4B, 5). However, the same effect was not observed in HCT116 cells, suggesting that the mechanisms involved in ATM mediated stabilization of HIF-1α is cell type specific. Contradicting results regarding ATM involvement in regulating HIF-1α stability has been previously reported (Cam et al., 2010; Ousset et al., 2010), which supports a cell type specific effect. A more detailed analysis of the correlation between the ATM and HIF-1 pathway is needed to shed light to these observations.

Increased MDM2 protein levels were seen following Ku55933 treatment in hypoxic conditions (Figure 5C), suggesting that ATM is involved in regulating MDM2 protein levels in hypoxia. To test MDM2 involvement in regulating Suv39H1 stability in hypoxia, siRNA was used to reduce MDM2 expression (Figure 5D). Knockdown of MDM2 increased Suv39H1 and p53 protein levels under hypoxic conditions (Figure 5E). Together, these results suggest that the presence of catalytically active ATM in hypoxia, leads to the upregulation of Suv39H1 by negatively regulating MDM2.

## Discussion

Our results provide direct evidence demonstrating that hypoxic activation of ATM requires the presence of H3K9me3 and Tip60 activity. This adds additional complexity to the previous reports of ATM activation in hypoxia as a consequence of replication stress (Olcina et al., 2013). Silencing Suv39H1 expression led to a significant decrease in the levels of H3K9me3, demonstrating that Suv39H1 plays an essential role in the induction of H3K9me3 in hypoxia. The importance of Suv39H1 as part of the cellular response to hypoxic stress is emphasized by the involvement of HIF-1α in inducing the expression of methionine adenosyltransferase 2A (Mat2A) (Liu et al., 2011). Mat2A regulates the homeostasis of the universal methyl donor S-adenosylmethionine (SAM) which functions as the methyl donor for Suv39H1 catalytic reactions (Müller et al., 2016). As such SAM promotes Suv39H1 activity, and hence the induction of H3K9me3, in response to hypoxic stress.

Here we show that Suv39H1 upregulation in hypoxia is a process regulated at the protein level by MDM2, supporting previous work (Bosch-Presegue et al., 2011; Mungamuri et al., 2016). In normoxic conditions, MDM2 dependent ubiquitination of Suv39H1 in response to oxidative stress is executed in a manner involving Sirt1 (Bosch-Presegue et al., 2011). However, MDM2 levels were unaffected and Sirt1 levels downregulated in hypoxia, suggesting an alternative mechanism regulating MDM2 activity in this setting.

Existing data highlights that the direct interaction of Tip60 with H3K9me3 is essential for the activation of ATM in response to DNA damage (Sun et al., 2005, 2007, 2009). This notion together with observed upregulation of Tip60 (Figure 3D) led us to test if hypoxic ATM activation required Tip60 activity. The data provided in this study supports this concept, as Tip60 inhibition abolished ATM autophosphorylation. Additionally, this is substantiated by the downregulation of Sirt1 (Figure 3C), as Sirt1 is involved in negatively regulating Tip60 activity (Wang and Chen, 2010; Peng et al., 2012). Sirt1 has been implicated in negatively regulating HIF-1α activity (Lim et al., 2010; Yu et al., 2019), which is further supported by the presented data. Together our results suggest that ATM activation in hypoxia is Tip60 dependent, expanding the previously proposed model indicating replication stress as the triggering event of ATM activation in hypoxia (Hammond et al., 2002; Olcina et al., 2013).

ATM has known roles in promoting heterochromatin formation (Filipponi et al., 2016), adjusting MDM2 activity (Cheng et al., 2009; Gannon et al., 2012) and protein stability (Khoronenkova et al., 2012) in response to DNA damage. In addition, ATM is known to be catalytically active in hypoxia, independently of DNA damage (Bencokova et al., 2009; Olcina et al., 2013). Therefore it was hypothesized that inhibition of MDM2 and consequent upregulation of Suv39H1 in hypoxia might be coordinated by ATM. Supporting this, the inhibition of ATM resulted in MDM2 upregulation, and significant downregulation of Suv39H1 protein levels in hypoxia. In addition, silencing MDM2 expression in hypoxia induced upregulation of Suv39H1, an effect that was eliminated in cells treated with the ATM inhibitor.

**FIGURE 6:**
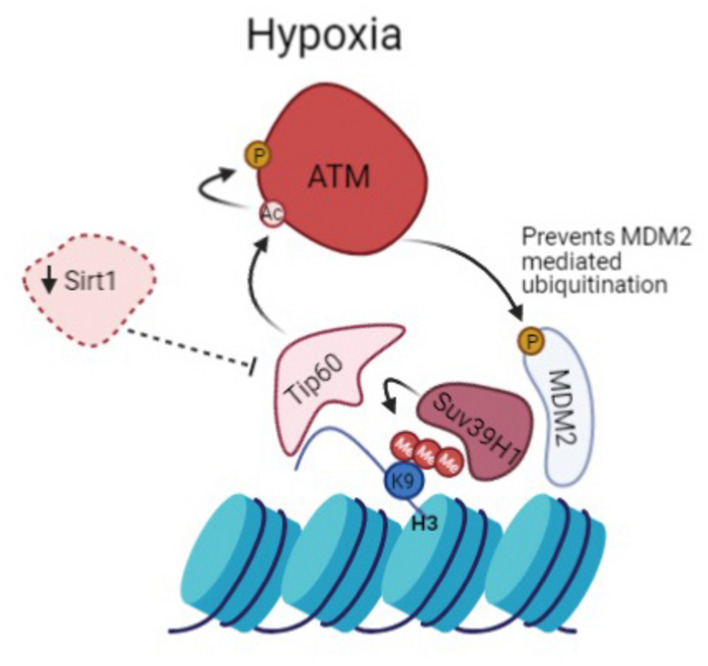
Prolonged activation of ATM in hypoxia promotes heterochromatin formation. Suv39H1 regulates H3K9me3 induction under hypoxia in a MDM2 dependent manner. Hypoxia activates Tip60, enabled by the downregulation of Sirt1. This leads to activation of ATM which negatively regulates MDM2 activity and protein levels allowing upregulation of Suv39H1 and maintenance of H3K9me3 giving rise to a novel positive feedback mechanism.

We propose that persistence of hypoxic conditions leads to sustained activation of ATM that directly regulates MDM2. This leads to the upregulation of Suv39H1 that helps maintain the methylation of H3K9, creating a positive feedback loop (Figure 6). This idea is further endorsed by data published by Ayrapetov et al. (2014) that shows that ATM dependent DDR activation is inhibited upon Suv39H1 knockdown (Ayrapetov et al., 2014). Furthermore, additional data shows that ATM activation requires Suv39H1 recruitment to chromatin to promote H3K9me3 and Tip60 activation (Qin et al., 2020).

## Conclusion

In conclusion, the data presented in this study highlight a previously uncharacterized feedback regulatory loop under hypoxic conditions, and point to a more complex role for ATM in determining cell fate under low oxygen conditions. The results presented here endorse the notion that the prolonged activation of ATM in hypoxia promotes heterochromatin formation. Specifically the ATM-MDM2 axis is involved in the regulation of Suv39H1 protein stability and enzymatic activity promoting H3K9me3. Epigenetic modifications of H3K9 have been associated with ATM activity in different cellular contexts including hypoxia (Xu et al., 2012; Olcina et al., 2013; Meyer et al., 2016). These findings advance our understanding of the pathways used by cancer cells to adapt to hypoxia and provide the platform for the design of novel potential therapeutic targets. The importance of this is emphasized by the increasing number of drugs targeting the DDR that are currently in different stages of development (Huang and Zhou, 2020). Particularly, the use of an ATM inhibitor, as a radiosensitizer, in malignancies known to have high levels of hypoxia, such as glioblastoma (Monteiro et al., 2017), has shown striking results *in vivo* (Durant et al., 2018).

## Data Availability Statement

The raw data supporting the conclusions of this article will be made available by the authors, without undue reservation.

## Author Contributions

ML: performed the experiments, data analysis, and drafted the manuscript. RGG, CD, KJW, and JB: experimental design, data analysis, and manuscript preparation. KJW: final approval of manuscript. All authors contributed to the article and approved the submitted version.

## Conflict of Interest

The authors declare that the research was conducted in the absence of any commercial or financial relationships that could be construed as a potential conflict of interest.

## Publisher’s Note

All claims expressed in this article are solely those of the authors and do not necessarily represent those of their affiliated organizations, or those of the publisher, the editors and the reviewers. Any product that may be evaluated in this article, or claim that may be made by its manufacturer, is not guaranteed or endorsed by the publisher.

## Supplementary Material

Supplementary Figure 1 Antibody validation. The specificity of two different MDM2 antibodies (anti-MDM2 EP16627 and anti-MDM2 2A10) was validated in cells treated with MDM2 siRNA showing significant reduction of the MDM2 band in cells transfected with siRNA targeting MDM2 expression. FTC133 cells were transfected with 40 pmol of MDM2 siRNA or control siRNA and analyzed by Western blot.

Supplementary Figure 2 ATM is activated in hypoxia in the absence of DNA damage. Cells were incubated in normoxia (N; 21% O_2_) or severe hypoxia (H; 0.1% O_2_) for 18 h prior to lysis and Western blotting. HIF-1α was used as a control for hypoxia and β-actin as a loading control **(A)** FTC133 cells were incubated for 18 h in normoxic (21% O_2_) or severe hypoxic (0.1% O_2_) conditions prior to fixation and staining. Cells irradiated with 4Gy cells were used as positive control (Nrmx + 4 Gy). Cells were stained for pATM-S1981 (green), 53BP1 (red) and DAPI (blue) **(B)**.

Supplementary Figure 3 The levels of ATM-pSer1981 in irradiated FTC133 cells treated with different concentrations of TH1834. Cells were incubated with 10, 20, or 30 μM of TH1834 or DMSO (marked with 0) in normoxia (21% O_2_) for 18 h and then irradiated at 4 Gy x-rays (N + 4Gy). Cells were lysed and analyzed by Western blot 1 h post radiation.

Supplementary Figure 4 Effect of ATM inhibition on the levels of Suv39H1 and MDM2 in normoxia. Cells were seeded left to adder overnight and incubated in normoxia (N: 21% O_2_) with DMSO or 10 μM of Ku55933 for 4 h prior to lysis and Western blot analysis.
