# Supplementary figures and images for: A Novel Mechanism of Ataxia Telangiectasia Mutated Mediated Regulation of Chromatin Remodeling in Hypoxic Conditions
Source: Front Cell Dev Biol. 2021 Sep 21;9:720194. doi: 10.3389/fcell.2021.720194 (PMC8491615; doi:10.3389/fcell.2021.720194)

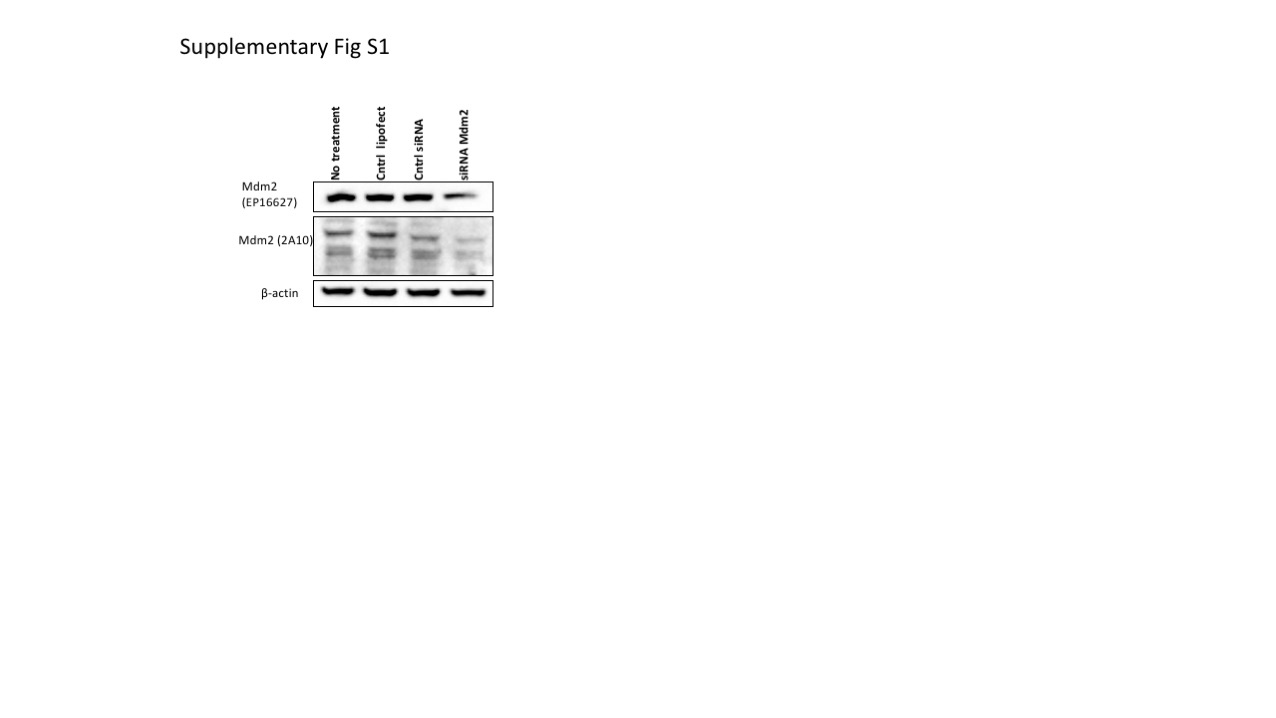

Supplement: Supplementary Figure 1 — Antibody validation. The specificity of two different MDM2 antibodies (anti-MDM2 EP16627 and anti-MDM2 2A10) was validated in cells treated with MDM2 siRNA showing significant reduction of the MDM2 band in cells transfected with siRNA targeting MDM2 expression. FTC133 cells were transfected with 40 pmol of MDM2 siRNA or control siRNA and analyzed by Western blot. [file Image_1.JPEG]

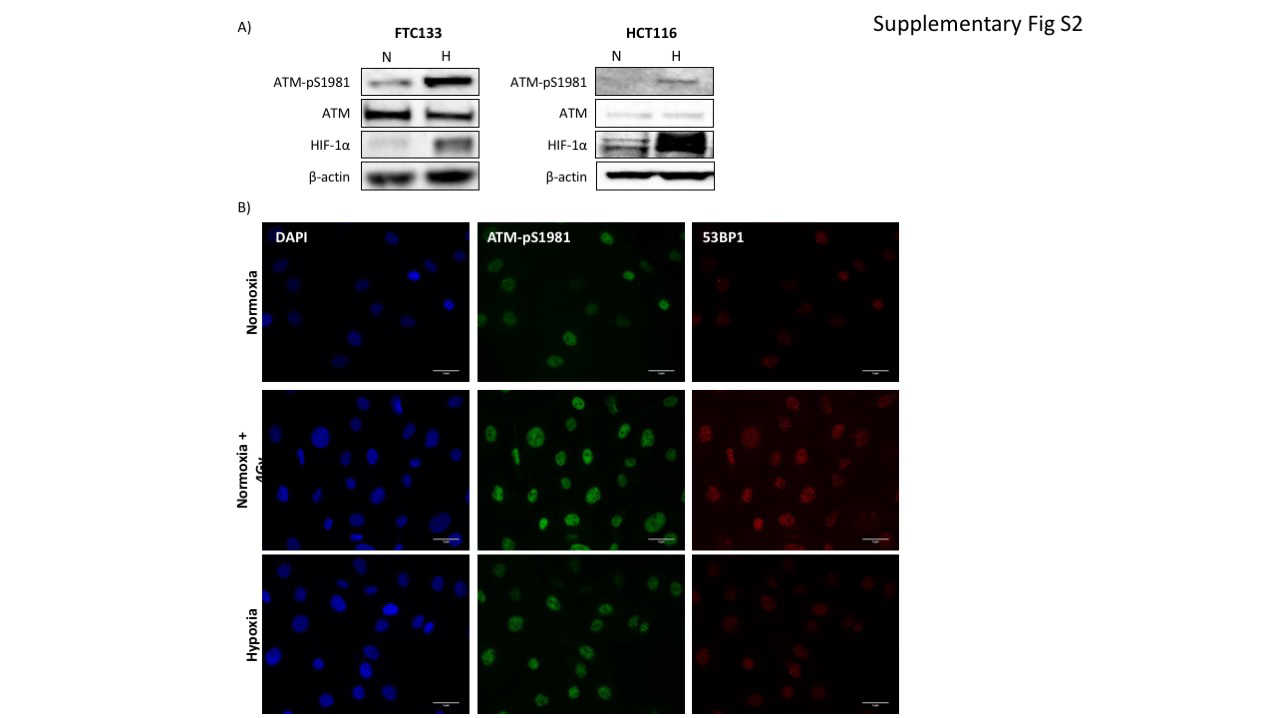

Supplement: Supplementary Figure 2 — ATM is activated in hypoxia in the absence of DNA damage. Cells were incubated in normoxia (N; 21% O2) or severe hypoxia (H; 0.1% O2) for 18 h prior to lysis and Western blotting. HIF-1α was used as a control for hypoxia and β-actin as a loading control (A) FTC133 cells were incubated for 18 h in normoxic (21% O2) or severe hypoxic (0.1% O2) conditions prior to fixation and staining. Cells irradiated with 4Gy cells were used as positive control (Nrmx + 4 Gy). Cells were stained for pATM-S1981 (green), 53BP1 (red) and DAPI (blue) (B). [file Image_2.JPEG]

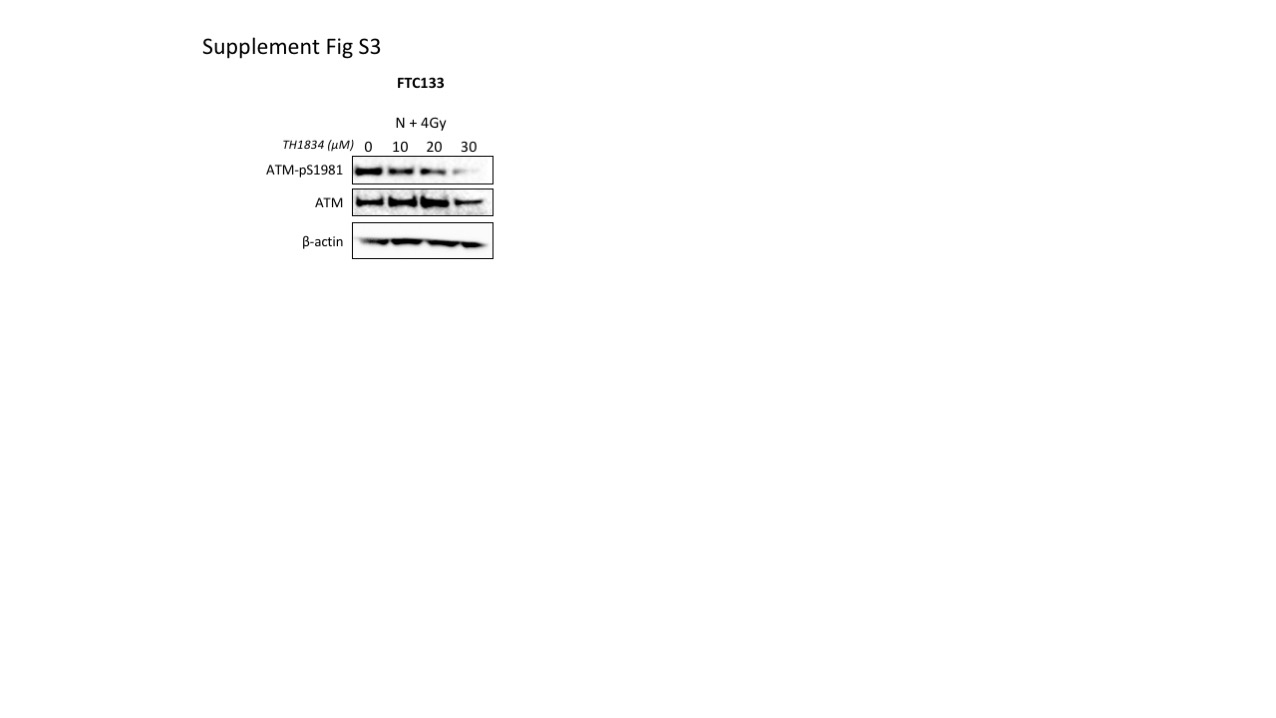

Supplement: Supplementary Figure 3 — The levels of ATM-pSer1981 in irradiated FTC133 cells treated with different concentrations of TH1834. Cells were incubated with 10, 20, or 30 μM of TH1834 or DMSO (marked with 0) in normoxia (21% O2) for 18 h and then irradiated at 4 Gy x-rays (N + 4Gy). Cells were lysed and analyzed by Western blot 1 h post radiation. [file Image_3.JPEG]

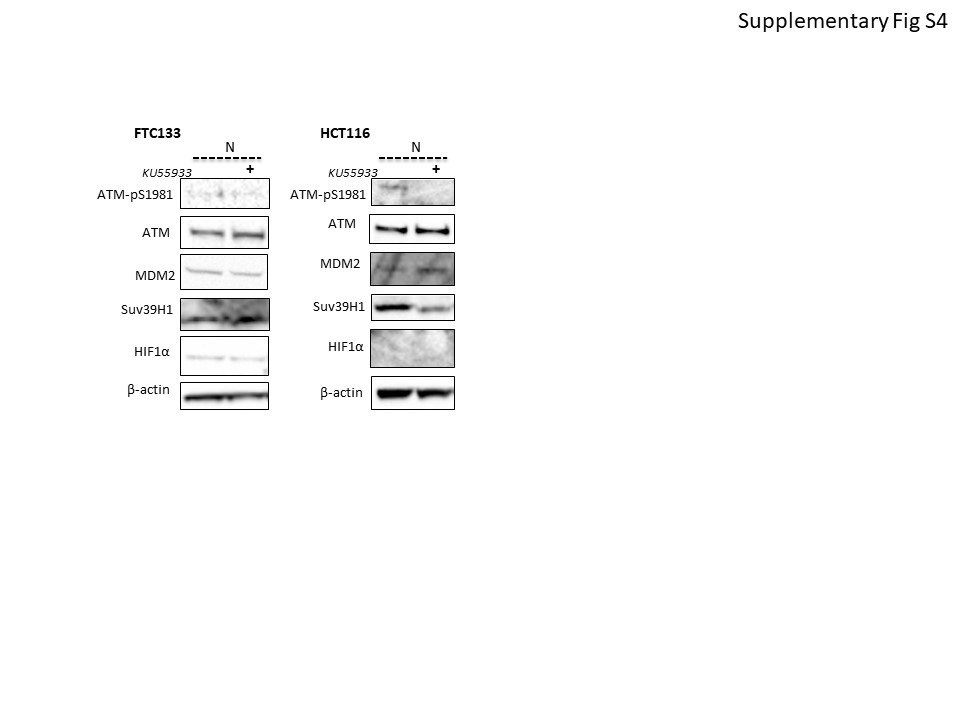

Supplement: Supplementary Figure 4 — Effect of ATM inhibition on the levels of Suv39H1 and MDM2 in normoxia. Cells were seeded left to adder overnight and incubated in normoxia (N: 21% O2) with DMSO or 10 μM of Ku55933 for 4 h prior to lysis and Western blot analysis. [file Image_4.JPEG]
